# Stat3 Inhibition Attenuates Mechanical Allodynia through Transcriptional Regulation of Chemokine Expression in Spinal Astrocytes

**DOI:** 10.1371/journal.pone.0075804

**Published:** 2013-10-03

**Authors:** Xiaodong Liu, Yuanyuan Tian, Na Lu, Tony Gin, Christopher H. K. Cheng, Matthew T. V. Chan

**Affiliations:** 1 Department of Anaesthesia and Intensive Care, The Chinese University of Hong Kong, Shatin, New Territories, Hong Kong Special Administrative Region, China; 2 School of Biomedical Sciences, The Chinese University of Hong Kong, Shatin, New Territories, Hong Kong Special Administrative Region, China; Universite de Rennes 1, France

## Abstract

**Background:**

Signal transducer and activator of transcription 3 (Stat3) is known to induce cell proliferation and inflammation by regulating gene transcription. Recent studies showed that Stat3 modulates nociceptive transmission by reducing spinal astrocyte proliferation. However, it is unclear whether Stat3 also contributes to the modulation of nociceptive transmission by regulating inflammatory response in spinal astrocytes. This study aimed at investigating the role of Stat3 on neuroinflammation during development of pain in rats after intrathecal injection of lipopolysaccharide (LPS).

**Methods:**

Stat3 specific siRNA oligo and synthetic selective inhibitor (Stattic) were applied to block the activity of Stat3 in primary astrocytes or rat spinal cord, respectively. LPS was used to induce the expression of proinflammatory genes in all studies. Immunofluorescence staining of cells and slices of spinal cord was performed to monitor Stat3 activation. The impact of Stat3 inhibition on proinflammatory genes expression was determined by cytokine antibody array, enzyme-linked immunosorbent assay and real-time polymerase chain reaction. Mechanical allodynia, as determined by the threshold pressure that could induce hind paw withdrawal after application of standardized von Frey filaments, was used to detect the effects of Stat3 inhibition after pain development with intrathecal LPS injection.

**Results:**

Intrathecal injection of LPS activated Stat3 in reactive spinal astrocytes. Blockade of Stat3 activity attenuated mechanical allodynia significantly and was correlated with a lower number of reactive astrocytes in the spinal dorsal horn. *In vitro* study demonstrated that Stat3 modulated inflammatory response in primary astrocytes by transcriptional regulation of chemokine expression including Cx3cl1, Cxcl5, Cxcl10 and Ccl20. Similarly, inhibition of Stat3 reversed the expression of these chemokines in the spinal dorsal horn.

**Conclusions:**

Stat3 acted as a transcriptional regulator of reactive astrocytes by modulating chemokine expression. Stat3 regulated inflammatory response in astrocytes and contributed to pain modulation. Blockade of Stat3 represents a new target for pain control.

## Introduction

Signal transducer and activator of transcription 3 (Stat3) is a member of the Stat transcription factor family that regulates gene expression in response to extracellular signals. These signals, including cytokines and growth factors, bind to their receptors and lead to the phosphorylation of Stat3 on tyrosine 705 through the receptor associated kinases, typically the Janus kinases (Jak). The phosphorylation in turn induces nuclear translocation and DNA binding that results in transcription of other genes. In this regard, genes that are involved in proliferation, anti-apoptosis and angiogenesis have been recognized as the principle targets of Stat3 [1]. Alternatively, phosphorylated Stat3 regulates gene expression through binding to other transcription factors such as nuclear factor kappa-light-chain-enhancer of activated B cells [2]. By regulating these target genes, Stat3 has been shown to increase cellular proliferation and augment inflammatory response [3].

Recent studies showed that proliferation of astrocytes along the pain pathways is a histological hallmark in chronic pain [4], [5]. Given that Stat3 regulates cellular proliferation [3], it is not surprising that it also modulates the development of chronic pain. In a recent study, the Jak-Stat3 pathway was activated in astrocytes and contributed to astrogliosis in a rat model of chronic pain following spinal nerve injury [6]. Similarly, blockade of the Jak-Stat3 pathway by Jak inhibitor mimicked the effects of cell cycle inhibition targeting at astrocyte proliferation and reduced chronic pain in rats [7].

Neuroinflammation has been observed in the development of chronic pain. Activated spinal astrocytes release proinflammatory chemokines (e.g. monocyte chemoattractant protein-1) and cytokines (e.g. interleukin 1 (IL-1) and IL-6) [5], [8] that are known to sensitize nociceptive transmission and contribute to the initiation and maintenance of chronic pain [9]. Although previous studies have shown that Stat3 regulates the expression of inflammatory genes [2], it is unclear whether Stat3 alters pain processing by regulating the inflammatory response in spinal astrocytes. In a rat model of chronic pain using intrathecal injection of endotoxin, we aimed at exploring the effect of Stat3 on neuroinflammation and the development of chronic pain.

## Materials and Methods

### Animal Model and Surgery

The experimental protocol and surgical procedures were approved by the Animal Experimentation Ethics Committee, The Chinese University of Hong Kong. We studied a rat model of mechanical allodynia produced by intrathecal injection of lipopolysaccharide (LPS) [9], [10]. Male Sprague Dawley (SD) rats weighing 300–350 g were used in the study. Following anesthesia with 3% isoflurane, the spinal column was exposed. A guiding needle (18 G) was passed between the lumbar vertebrae 5 and 6 to enter the intrathecal space. A PE10-polyethylene catheter was then implanted through the guiding needle until it reached the lumbar enlargement of the spinal cord, typically at a depth of 2 cm from the skin. The outer end of the catheter was plugged and was transfixed to the skin. The rats were housed individually after surgery and 7 days were allowed for recovery before further treatment.

### Reagents

We used Stattic (Tocris Bioscience, Bristo, UK), a selective Stat3 inhibitor [11], in our experiments. Stattic was dissolved in dimethyl sulfoxide (DMSO, Sigma-Aldrich; St Louis, MO) at a concentration of 50 mM and was stored at −20°C before use. LPS was derived from *E. coli* O111:B4 (Millipore, Billerica, MA), prepared at 1 mg/mL in phosphate buffered saline (PBS).

### Painful Behavioral Test

In all experiments, mechanical allodynia was determined using standard von Frey filaments (IITC Life Science, Woodland Hills, CA, USA). Briefly, a series of 10 von Frey filaments, with bending force of 0.4, 0.6, 1, 1.4, 2, 4, 6, 8, 10, 15, 26 and 60 g, were applied to the mid-plantar surface of the hind paws. A stimulus-related withdrawal of the tested paw was considered a positive withdrawal response. The paw withdrawal threshold (PWT) was determined by the mean of the two filaments resulting just in a positive and a negative withdrawal response using the Dixon’s up-and-down technique [12].

### Primary Astrocyte Culture

Primary astrocytes were isolated and purified from the cortex of neonatal (1–2 days old) SD rats. Rat pups were euthanized using pentobarbital overdose. The cortex was collected, cut into pieces and suspended in 3 mL digestive solution containing 0.25% trypsin-EDTA (Invitrogen; Life Technologies, Carlsbad, CA) and 50 µg/mL of DNase I (Hoffmann-La Roche; Basel, Switzerland). After incubation at 37°C for 15 min, cells were collected by pipetting up and down gently, followed by centrifugation at 1,500 rpm for 10 min. Cells were then re-suspended and seeded onto 75 cm^2^ flasks and grew to confluency. Astrocytes were purified by shaking (speed = 225 rpm) at 37°C for 8 hours. Purified astrocytes were grown in Dulbecco’s modified Eagle medium (DMEM) with 10% heat-inactivated fetal bovine serum (Invitrogen; Life Technologies, Grand Island, NY) and 1% antimyotic at 37°C.

### Immunofluorescence

Goat anti-glial fibrillary acidic protein (GFAP) and rabbit anti-Stat3 (Santa Cruz Biotechnology, Santa Cruz, CA) were used. Rabbit anti-phospho-Stat3 (pTyr705) was obtained from Cell Signaling Technology (Beverly, MA), whereas Alexa 488 conjugated anti-goat and Alexa 555 conjugated anti-rabbit antibodies were obtained from Molecular Probes (Invitrogen; Life Technologies, Grand Island, NY). For cell staining, astrocytes were seeded on glass slides. Cells were fixed with 4% paraformaldehyde (PFA) in PBS for 15 min, and then rinsed (three times, 5 min each) in PBS. For staining of GFAP, cells were blocked in blocking buffer containing 5% normal goat serum and 0.3% Triton X-100 in PBS for 1 hour at room temperature. For staining of Stat3, an additional permeabilization step by ice-cold 100% methanol (incubated for 10 min at −20°C) was done before blocking. After blocking, cells were incubated for overnight in primary antibody solution (1 µg/mL for anti-GFAP and 2 µg/mL for anti-Stat3, prepared in PBS containing 1% bovine serum albumin (BSA) and 0.3% Triton X-100). After rinsing in PBS (three times, 5 min each), cells were incubated in fluorochrome-conjugated secondary antibodies (Molecular Probes, diluted 1∶500 in PBS containing 1% BSA and 0.3% Triton X-100) for 1 hour at room temperature. Cells were then rinsed in PBS (three times, 5 min each) and mounted in ProLong Gold Antifade Reagent with 4′,6-diamidino-2-phenylindole.

A similar protocol for staining of spinal cord sections was adopted. Fresh lumbar spinal cords were harvested and fixed with 4% PFA for 24 hours at 4°C. Spinal cords were dehydrated in a series of sucrose solutions (10%, 20% and 30% of sucrose in 4% PFA PBS). The spinal cords were then embedded in optimal cutting temperature compound (Sakura Finetek; Torrance, CA) and 25 µm sections were cut on a cryostat (Leica, Nussloch, Germany). All the staining steps were the same as the above mentioned immunofluorescence methods, except that the two primary antibodies (anti-GFAP at 2 µg/mL and anti-phospho-Stat3, at 1∶80 dilution) or the two secondary antibodies (at 1∶500 dilution) were prepared in PBS containing 1% BSA and 0.3% Triton X-100. All images were collected on a Zeiss laser scanning microscope (Carl Zeiss; Germany) using 20× objective with the same microscope acquisition parameters.

### RNA Interference

Small interfering RNA oligo (si-Stat3) against rat Stat3 was designed and synthesized by RIBOBIO (Guangzhou, China). We used siRNA that did not target any rat gene as negative control (si-Neg). A final concentration of 20 µM for each siRNA oligo was transfected into the cultured cell using Lipofectamine 2000 (Invitrogen; Life Technologies).

### 3(4,5-dimethylthiazol-2-yl)-2,5-diphenyltetrazolium Bromide (MTT) Assay

The astrocyte cell number was determined after siRNA oligo transfection using MTT assay. Briefly, astrocytes were seeded into 96-well plates (3,000 cells/well). After transfection with siNeg or siStat3 oligos for 48 hours, 20 µL of MTT solution (5 mg/mL in PBS) were added into each well and incubated at 37°C for another 1 hour. The supernatants were then removed and the remaining formazan crystals dissolved in 100% DMSO. The spectrophotometric absorbance of each well was determined at a wavelength of 570 nm.

### Cytokine Antibody Array

The rat cytokine array ARY008 (R&D Systems, Minneapolis, MN) was employed. Protein samples were prepared in a cytokine array lysis buffer (1% NP-40, 20 mM Tris, 137 mM NaCl, 10% glycerol, pH 8.0) containing 10% protease inhibitor cocktail (Hoffmann-La Roche). The protein samples (150 µg of protein for each sample) were mixed with cytokine/chemokine detection antibodies cocktail and incubated for 1 hour at room temperature. The mixtures were then incubated with a blot array precoated with 29 cytokine/chemokine capture antibodies for another 12 hours at 4°C. The blot arrays were then incubated with Streptavidin-HRP for 30 min at room temperature and visualized using an enhanced chemiluminescence detection system and hyperperformance chemiluminescence film (GE Healthcare, Little Chalfont, UK). The density of each dot was analyzed in Adobe Photoshop CS3 (Adobe Systems, San Jose, CA).

### Enzyme-linked Immunosorbent Assay (ELISA)

Cytokine release was quantified using ELISA methods. Supernates from cell culture were collected from dishes and were centrifuged (3,000 rpm at 4°C for 10 min) before measurements. Protein levels for Cxcl2 and Timp-1 were measured using rat ELISA kits (R&D Systems, Minneapolis, MN). Similarly, specific rat ELISA kits were used for Cx3cl1, Cxcl5 (Abcam, Cambridge, MA), Ccl3, Ccl20 and Cxcl10 measurements (Abnova, Taoyuan, Taiwan). All assays were performed according to the instructions of the manufacturers.

### Quantitative Real Time PCR (qRT-PCR)

Total RNA from primary cells or tissues were extracted using Trizol/Phenol/Chloroform following a standard protocol. Complementary DNA (cDNA) was reversed from 2 µg of total RNA using high capacity cDNA reverse transcription kit. The levels of each gene were determined by SYBR green master mixture on HT7900 system (Applied Biosystems; Life Technologies), with β-actin as the internal control. Primers used in this study are listed in the Table 1.

**Table 1:** Primer sequences for qRT-PCR.

Gene	Forward (5′ to 3′)	Reverse (5′ to 3′)
β-actin	CACCCGCGAGTACAACCTTC	CCCATACCCACCATCACACC
Cx3cl1	GGTGGCAAGTTTGAGAAGCG	CCTGGGAAATAGCAGTCGGT
Cxcl5	TGGCATTTCTGCTGCTGTT	TGCATTCCGCTTTGTTTTC
Cxcl10	CTGCACCTGCATCGACTTCC	TTCTTTGGCTCACCGCTTTC
Ccl20	TACTGCTGGCTTACCTCTGC	CACGGATCTTTTCGACTTCA
Il-1α	GAGTGCTCAGGGAGAAGACAAG	TCTGGAAATCTATCATGGAGGG
iNos	CAGCCCTCAGAGTACAACGAT	CAGCAGGCACACGCA ATGAT
Stat3	AGAGGCGGCAGCAGATAGC	TTGTTGGCGGGTCTGAAGTT

### Western Blot

Cells were lysed in lysis buffer (50 mM Tris, 150 nM NaCl, 1% NP-40, 1 mM EDTA, pH 7.6) containing 10% protease inhibitor cocktail. Total protein was collected and the concentration was measured by the Pierce BCA protein assay kit (Thermo Fisher, Rockford, IL). Protein samples were separated on 12% SDS-polyacrylamide gel and transferred onto a hybridization nitrocellulose membrane (0.45 µM) (Millipore, Billerica, MA). After blocking with 5% skim milk for 1 hour, membranes were incubated with primary antibodies against phospho-Stat3 (1∶1,000, Cell Signaling technology), Stat3 (1∶2,000, Santa Cruz) or β-actin (1∶3000, Santa Cruz) overnight at 4°C. The membranes were incubated with secondary antibody for 1 hour at room temperature. The protein bands were visualized using the enhanced chemiluminescence detection system (GE Healthcare Biosciences, Pittsburgh, PA).

### Statistical Analyses

Data are presented as mean values ± standard error of the mean (SEM). PWT from behavioral assessment were analyzed among groups using analysis of variance (ANOVA). Intergroup difference was tested using unpaired *t* test. Data from the MTT assays and qRT-PCR were compared between groups using unpaired *t* test. A *p* value <0.05 was considered statistically significant.

## Results

### Stat3 Contributed to LPS Induced Mechanical Allodynia by Regulating Reactive Astrocytes

**Figure 1:**
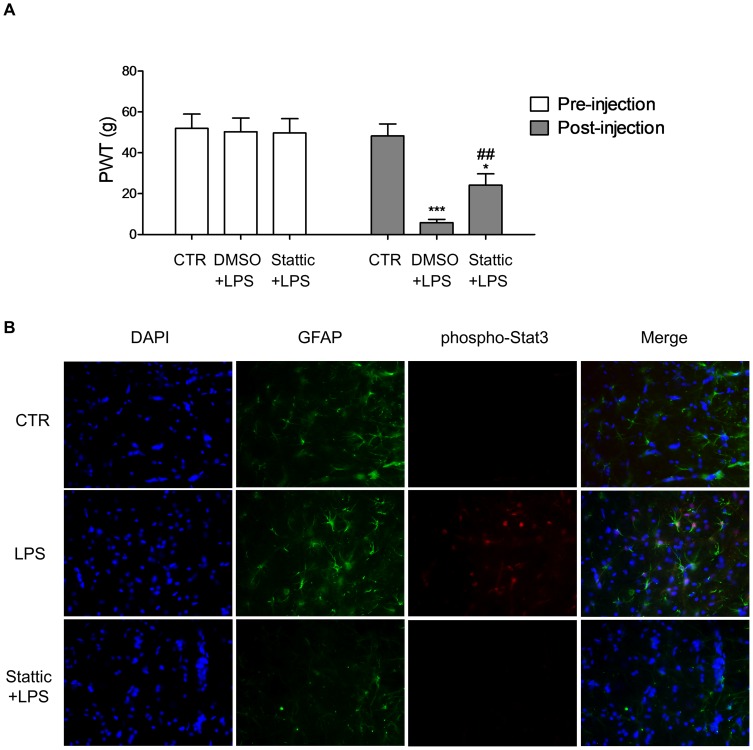
Stat3 contributes to LPS induced mechanical allodynia by regulating reactive astrocytes. A. Intrathecal LPS injection led to mechanical allodynia. Pretreatment with Stattic attenuated LPS induced allodynia. *n* = 8 in each group. *p<0.05; ***p<0.001 compared with control (CTR). ^##^ *p*<0.01 compared with DMSO+LPS, unpair *t* test. B. Intrathecal LPS injection enhanced staining of phospho-Stat3 in lumbar dorsal horn slices. Phospho-Stat3 immunoreactive cells were also co-labelled with GFAP. Pretreatment with Stattic reduced the immunoreactivity of GFAP and phospho-Stat3. Original magnification, 200×. Error bars represent SEM.

In this experiment, 24 rats were randomly assigned into three groups (*n* = 8 per group), *viz* vehicle control, DMSO+LPS and Stattic+LPS group. All animals received 2 intrathecal injections. In the first injection, animals in the control group and DMSO+LPS group received 10 µL of DMSO solution (1% DMSO in saline, a vehicle control for Stattic). Animals in the Stattic+LPS group received 10 µL of Stattic (50 µM in saline). One hour later, all animals received the second injection. In the control group, 10 µL of saline was administered. The remaining rats in the DMSO+LPS and the Stattic+LPS groups received 10 µL of LPS (1 mg/mL). Following intrathecal injection, LPS significantly reduced mechanical PWT (DMSO+LPS and Stattic+LPS groups) as compared with the controls (Figure 1A, *p*<0.001, ANOVA). Pretreatment with Stattic attenuated LPS induced mechanical allodynia. These data indicated that Stat3 is involved in LPS induced pain model.

Histological changes after the behavioral testing were also evaluated. Rats were euthanized for spinal cord harvest and subsequent immunofluorescence staining. As compared with the controls, LPS injection enhanced staining of phospho-Stat3 (Tyr 705) in the spinal dorsal horn isolated from the DMSO+LPS group (Figure 1B). The phospho-Stat3 cells were also double-labeled with GFAP (Figure 1B), indicating activated Stat3 in reactive astrocytes by the LPS injection. In contrast, pretreatment with Stattic reduced both phospho-Stat3 and GFAP staining in the spinal dorsal horn sections (Figure 1B). Taken together, these results showed that Stat3 activity regulated the proliferation of reactive astrocytes and contributed to pain modulation.

### LPS Activated Stat3 in Primary Astrocytes

**Figure 2:**
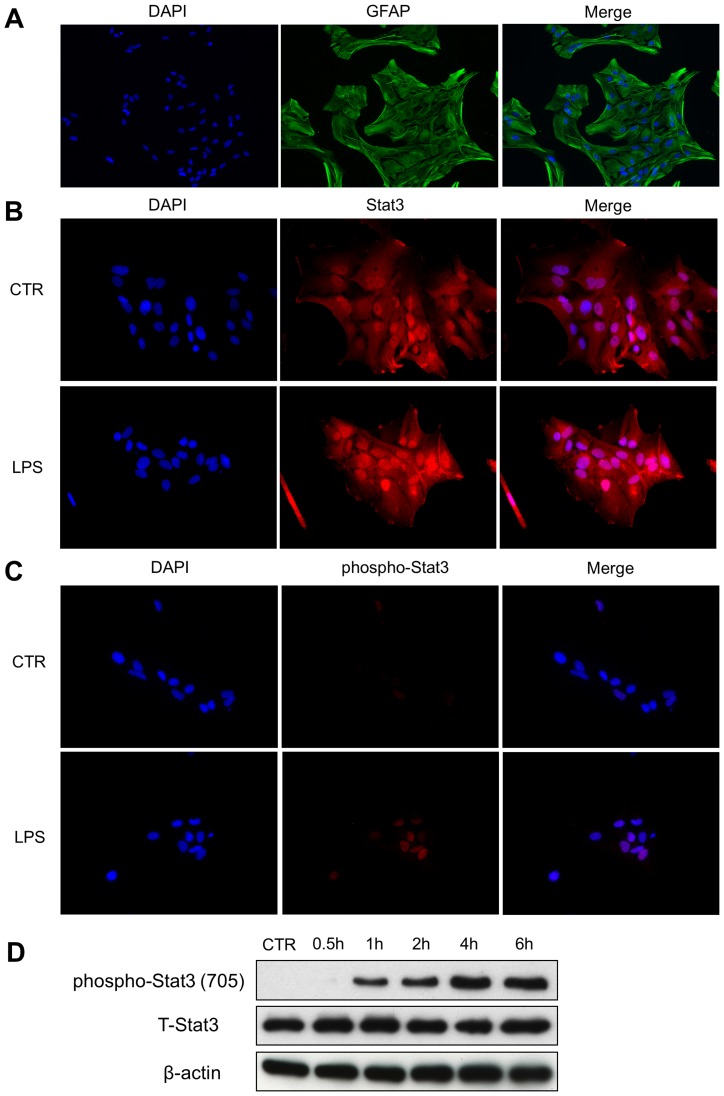
LPS activates Stat3 in primary cultured astrocytes. A. Primary astrocyte cultures. Over 99% cells were labeled with GFAP. B. Stat3 distribution in astrocytes. Stat3 was distributed in both nucleus and cytoplasm of the cultured astrocytes. LPS treatment did not further induce nuclear entry of Stat3. C. Phospho-Stat3 staining in astrocytes. LPS treatment for 2 hours enhanced phospho-Stat3 staining in the nucleus. D. Western blot analysis of phospho-Stat3. LPS treatment caused increased phosphorylation of Stat3 on Tyr 705 in a time-dependent fashion. Original magnification, 200×.

In order to investigate the regulation of Stat3 in reactive astrocytes, primary cultured astrocytes were purified from the cortex of neonatal rat pups. As confirmed by immunofluorescence staining, the purity of astrocytes was over 99% (Figure 2A). Stat3 was primarily distributed in both nucleus and cytoplasm. LPS treatment failed to induce nuclear entry of Stat3 (Figure 2B). However, LPS increased phospho-Stat3 (Tyr 705) staining in the nucleus (Figure 2C), suggesting that the action of LPS on Stat3 activation depends on post-translational modification but not on subcellular localization. Western blot also showed that LPS induced phosphorylation of Stat3 on Tyr 705 in a time-dependent fashion (Figure 2D). These results confirmed that LPS activated Stat3 in astrocytes.

### Blockade of Stat3 Reduced LPS Induced Chemokines in Astrocytes

**Figure 3:**
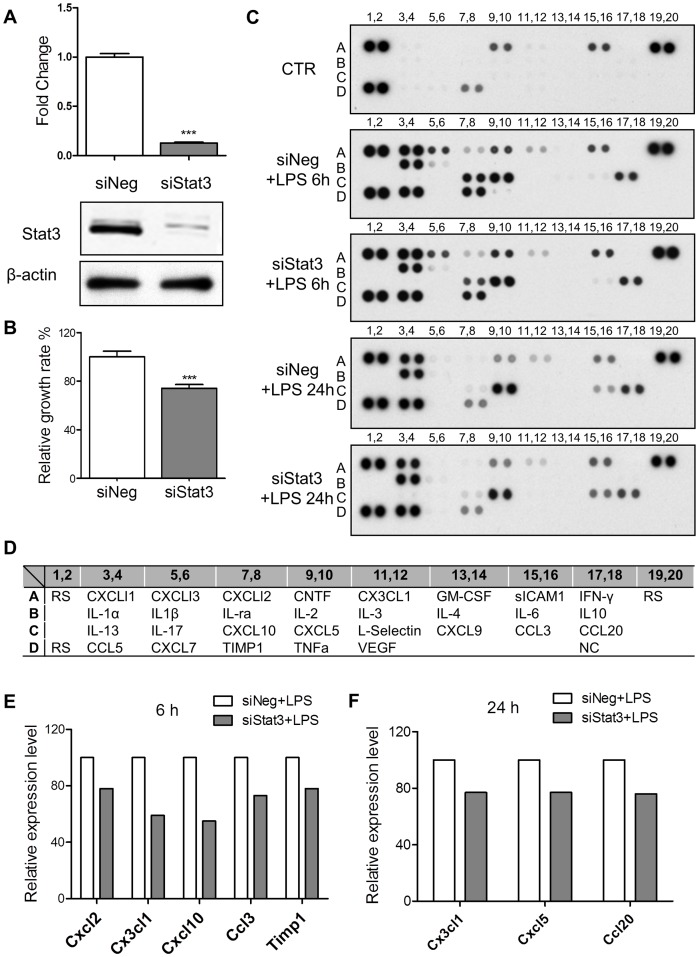
Stat3 was involved in LPS induced chemokines protein expression. A. Stat3 knockdown. siStat3 oligo transfection caused reduction of Stat3 mRNA and protein. *n* = 3, ***p<0.001, compared with siNeg, unpaired *t* test. B. siStat3 oligo transfection significantly decreased astrocyte cell number as determined by MTT assay. *n* = 3, ***p<0.001, compared with siNeg, unparied *t* test. C. Cytokine antibody array. LPS treatment for 6 hours led to up-regulation of at least 13 proteins including Il-1α (B3,4), Il-1β (B5,6), TNFa (D9,10), Cxcl1 (A3,4), Cxcl3 (A5,6), Cxcl (A7,8), Cx3cl1 (A11,12), Cxcl10 (C7,8), Cxcl5 (C9,10), Ccl3 (C15,16), Ccl20 (C17,18), Ccl5 (D3,4) and Timp1 (D7,8). siStat3 transfection reduced the effects of LPS, causing attenuated levels of Cxcl2, Cx3cl1, Cxcl10, Ccl3 and Timp1 compared to the siNeg transfected cells. LPS treatment for 24 hours led to up-regulation of at least 7 proteins, including Cxcl1, Cx3cl1, Il-1α, Cxcl5, Ccl3, Ccl20, Ccl5. Stat3 knockdown reduced LPS induced up-regulation of Cx3cl1, Cxcl5 and Ccl20. D. List of cytokines and chemokines in the antibody array. RS: reference spot, used as positive control; NC: negative control. E. Relative levels of differentially expressed proteins between siNeg+LPS and siStat3+LPS treated astrocytes (6 hours). siStat3 caused a reduction of LPS induced Cxcl2 (22% reduction), Cx3cl1 (41% reduction), Cxcl10 (45% reduction), Ccl3 (27% reduction) and Timp1 (22% reduction), as compared with the siNeg+LPS treatment. F. Relative levels of differentially expressed proteins between siNeg+LPS and siStat3+LPS treated astrocytes (24 hours). When normalized to siNeg+LPS treatment, Stat3 knockdown reduced LPS induced Cx3cl1 (23% reduction), Cxcl5 (23% reduction) and Ccl20 (24% reduction). Error bars represent SEM.

In this experiment, astrocytes were transfected with siNeg (negative control oligo) or siStat3 (Stat3 specific RNAi oligo) for 48 hours. The qRT-PCR results showed that the mRNA level of Stat3 was reduced to 13% of the baseline value by siStat3 as compared with siNeg (Figure 3A). This was confirmed by Western blot analysis (Figure 3A). Using a MTT assay after transfection, it was found that siStat3 transfection caused a significant reduction (26%, *n* = 3, *p*<0.001) of cell number as compared with siNeg (Figure 3B), confirming the action of siStat3 oligo.

Cytokine antibody array analysis was conducted to determine the effects of Stat3 blockade on the expression of proinflammatory genes. Prior to LPS treatment, astrocytes were transfected with either siNeg or siStat3 for 48 hours. Astrocytes were then treated with LPS (1 µg/mL) for 6 or 24 hours. The protein sample without LPS treatment was collected and used as vehicle control. After treatment for 6 hours, at least 13 differential proteins were up-regulated to different extents in the siNeg+LPS group as compared with the controls (Figure 3C). Among these proteins, tissue inhibitor of metalloproteinase 1 (Timp1) functions as an anti-inflammatory factor by blocking the activity of matrix metalloproteinases (Mmps). In contrast, the other proteins were reported to exert proinflammatory actions. Only 3 cytokines including IL-1α, IL-1β and tumor necrosis factor α (TNFα) were identified as LPS induced target proteins. The remaining targets i.e. chemokine (C_X_C motif) ligand 1 (Cxcl1), Cxcl3, Cxcl2, Cxcl10, Cxcl5, chemokine (C_X3_C motif) ligand 1 (Cx3cl1), chemokine (C_C motif) ligand 3 (Ccl3), Ccl20 and Ccl5 belong to the chemokine family. After 24 hours, at least 7 target proteins including Cxcl1, Cx3cl1, IL1α Cxcl5, Ccl3, Ccl20 and Ccl5 were up-regulated in siNeg+LPS treated astrocytes as compared with the control (Figure 3C). To investigate the effects of Stat3 blockade on these target proteins, the relative amounts of the differentially expressed target proteins between siNeg+LPS astrocytes and siStat3+LPS astrocytes was compared. When normalized to siNeg+LPS, it was found that siStat3 caused a relative reduction of LPS induced Cxcl2 (22% reduction), Cx3cl1 (41% reduction), Cxcl10 (45% reduction), Ccl3 (27% reduction), and Timp1 (22% reduction) at 6 hours (Figure 3E). In addition, siStat3 also down-regulated the level of Cx3cl1 (23% reduction), Cxcl5 (23% reduction) and Ccl20 (24% reduction) after treatment with LPS for 24 hours (Figure 3F). These findings confirmed that LPS is a potent inducer of chemokines and Stat3 acts as a downstream regulator of these chemokines.

**Figure 4:**
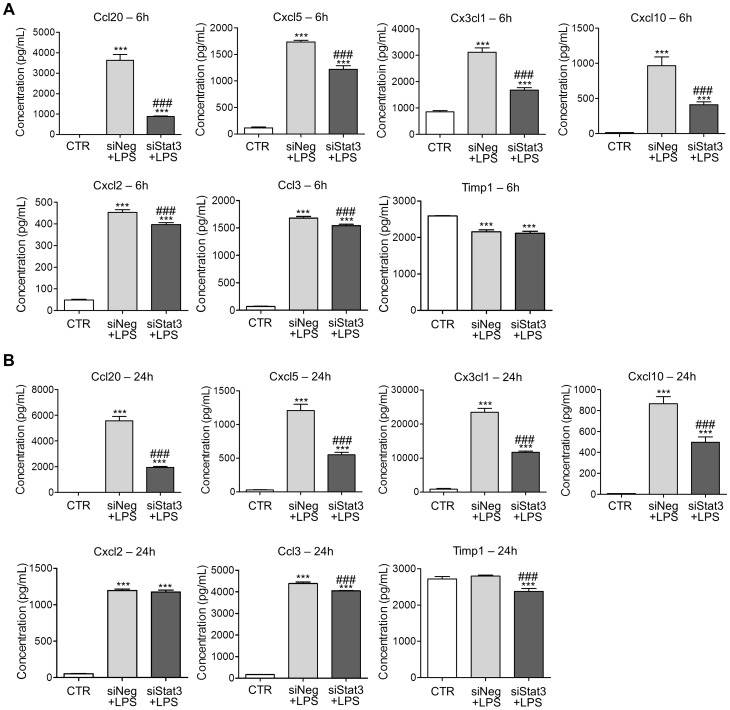
Stat3 knockdown reduced LPS induced chemokines protein level. A. Quantitative measurements of chemokines after treatment with LPS for 6 hours. Stat3 knockdown significantly reduced the expression of Ccl20 (75% reduction), Cxcl5 (30% reduction), Cx3cl1 (46% reduction) and Cxcl10 (57% reduction) after LPS treatment. In addition, Stat3 knockdown reversed the expression of Cxcl2 (12% reduction) and Ccl3 (11% reduction) modestly. LPS did not change Timp-1 protein level after LPS treatment for 6 hours. Similarly, siStat3 did not affect Timp-1 protein release. B. Quantitative measurements of chemokines after treatment with LPS for 24 hours. siStat3 significantly down-regulated the expression of Ccl20 (68% reduction), Cxcl5 (55% reduction), Cx3cl1 (49% reduction) and Cxcl10 (42% reduction) after LPS treatment. However, expression of Ccl3 (8% reduction) was only modestly reduced by siStat3 pretreatment and siStat3 has no effect on Cxcl2 expression. LPS treatment for 24 hours did not alter Timp-1expression, but siStat3 down-regulate Timp-1 by15%. *n* = 4 in all experiments, ***p<0.001, compared with control. ^###^ *p*<0.001, compared with siNeg+LPS, unpaired *t* test. Error bars represent SEM.

Furthermore, ELISA was performed to quantify the levels of target proteins that were identified in the cytokine array study. Following transfection of astrocytes, cell lysates or supernates from cell culture were collected for ELISA analyses after treatment with LPS for 6 or 24 hours. LPS treatment up-regulated all the chemokines except Timp-1 at both time points. LPS induced elevation of Ccl20, Cxcl5, Cx3cl and Cxcl10 levels were significantly reduced by Stat3 knockdown to different extents (Figure 4A and 4B). When normalized to siNeg+LPS, it was shown that siStat3 reduced the level of Ccl20 (75% reduction), Cxcl5 (30% reduction), Cx3cl1 (46% reduction) and Cxcl10 (57% reduction) at 6 hours after LPS treatment (Figure 4A). After treatment with LPS for 24 hours, siStat3 also down-regulated the expression of Ccl20 (68% reduction), Cxcl5 (55% reduction), Cx3cl1 (49% reduction) and Cxcl10 (42% reduction) (Figure 4B). In addition, siStat3 modestly altered the expression of Cxcl2 (12% reduction at 6 hours) and Ccl3 (11% and 8% reduction at 6 and 24 hours after LPS treatment, respectively) and Ccl3 after LPS treatment (Figure 4A and 4B). These findings confirmed that Stat3 acted as a modulator of LPS induced expression of chemokines, especially Ccl20, Cxcl5, Cx3cl1 and Cxcl10.

### Stat3 Contributed to Chemokine Expression via Transcriptional Regulation

**Figure 5:**
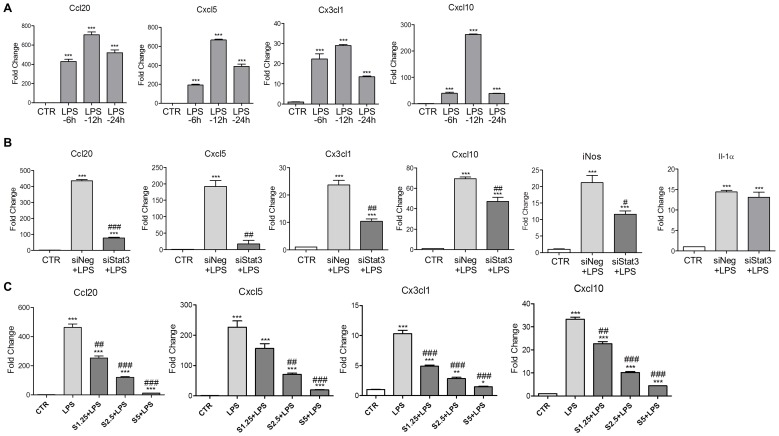
Stat3 transcriptionally regulated LPS induced chemokines. A. LPS caused significantly up-regulation of Ccl20, Cxcl5, Cx3cl1, and Cxcl10 in a time dependent manner. *n* = 3, ***p<0.001, compared with control (CTR), unpaired *t* test. B. Stat3 knockdown significantly reversed LPS induced Ccl20, Cxcl5, Cx3cl1, Cxcl10. As a positive control, the expression of iNos expression in astrocytes was also reversed by Stat3 knockdown. Similar with results of the cytokine array, the mRNA level of Il-1α was not affected by siStat3 transfection. *n* = 3, ***p<0.001, compared with control. ^#^ *p*<0.05; ^##^ *p*<0.01; ^###^ *p*<0.001, compared with siNeg+LPS, unpaired *t* test. C. Stattic repressed LPS induced chemokine expression in a dose dependent manner. *n* = 3, *p<0.05; **p<0.01; ***p<0.001, compared with control; ^##^ *p*<0.01; ^###^ *p*<0.001, compared with LPS, unpaired *t* test; S1.25, S2.5 and S5 represent pretreatment with Stattic at concentrations of 1.25 µM, 2.5 µM and 5 µM, respectively. Error bars represent SEM.

In the following experiments, the effects of LPS on the mRNA expression of differential chemokines were investigated. LPS up-regulated the mRNA level of Cx3cl1, Cxcl10, Cxcl5 and Ccl20 in a time-dependent fashion (Figure 5A), indicating the up-regulation of chemokines in the LPS treated astrocytes was due to enhanced transcription. The activity of Stat3 was then blocked by RNAi. Astrocytes were transfected with siNeg or siStat3 for 48 hours and then treated with LPS (1 µg/mL) for 6 hours. LPS up-regulated all these four chemokines in astrocytes transfected with siNeg (Figure 5B). Transfection of siStat3, however, significantly reduced the effects of LPS (Figure 5B). Compared with siNeg, knockdown of Stat3 reduced the expression of Cx3cl1 (56% reduction), Cxcl10 (32% reduction), Cxcl5 (91% reduction) and Ccl20 (83% reduction). The Stat3 activity was further blocked by Stattic. Prior to LPS treatment, astrocytes were treated with Stattic for 1 hour in three doses (1.25, 2.5 and 5 µg/mL). The qRT-PCR results revealed that Stattic significantly reduced LPS dependent up-regulation of these four chemokines in a dose-dependent fashion (Figure 5C).

In addition, the effects of siStat3 on inducible nitric oxide synthase (iNos) and IL-1α expression were also demonstrated (Figure 5B). LPS up-regulated the mRNA level of iNos, and Stat3 RNAi reversed the effects of LPS, causing a 45% reduction in mRNA level. In contrast, Stat3 RNAi failed to reverse the up-regulation of IL-1α mRNA. This data is consistent with the results in the cytokine array study (Figure 3C). Given that Stat3 binding elements are found in the promoter regions of iNos but not in IL-1α, these results revealed that Stat3 conceivably functions as a direct transcriptional factor of chemokine expression in LPS induced reactive astrocytes. It should be noted that among these chemokines, Cx3cl1 and Cxcl5 were identified as novel target genes of Stat3. Importantly, given that several of these chemokines especially Cx3cl1 are involved in nociception, our data suggested that the action of Stat3 on LPS induced mechanical allodynia probably acts through regulating chemokine expression.

### Stat3 Contributed to Chemokine Expression in Spinal Dorsal Horn

**Figure 6:**
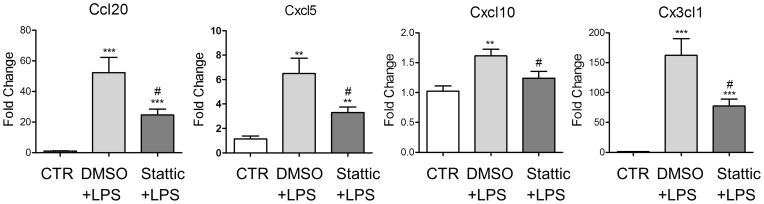
Stat3 regulation of chemokine expression is correlated with LPS induced mechanical allodynia. A. Intrathecal LPS injection up-regulated chemokine expression in the spinal dorsal horn. Pretreatment with Stattic partially reversed LPS induced chemokines up-regulation. *n* = 6 in each group, **p<0.01; ***p<0.001, compared with control, unpaired *t* test. ^#^ *p*<0.05, compared with DMSO+LPS, unpaired *t* test. Error bars represent SEM.

In order to evaluate the role of Stat3 in LPS induced chemokine expression *in vivo*, LPS or Stattic was injected intrathecally as above. The lumbar dorsal horns were separated and used to detect the mRNA level of chemokines identified above. Similar to our findings in primary astrocytes, chemokines including Cx3cl1, Cxcl10, Cxcl5 and Ccl20 were significantly up-regulated in the DMSO+LPS treated rats (*n* = 6) as compared with the control group (*n* = 6) (Figure 6). In contrast, pretreatment with Stattic (*n* = 6) significantly reduced the mRNA level of Cx3cl1 (52% reduction), Cxcl10 (23% reduction), Cxcl5 (45% reduction) and Ccl20 (53% reduction) as compared with the DMSO+LPS treated rats (*n* = 6). Our data suggested that suppression of these chemokines by blockade of Stat3 could attenuate pain-like behavior in neuroinflammation-dependent pain.

## Discussion

In this study, we have shown that intrathecal LPS produced mechanical allodynia by activating Stat3 in spinal astrocytes. Given that pretreatment with Stattic not only blocked the phosphorylation of Stat3 but also reduced GFAP staining, our data suggest that Stat3 inhibition is sufficient to decrease reactive spinal astrogliosis.

Recent studies demonstrated the involvement of astrocytic Jak-Stat3 pathway in the regulation of neuropathic pain [13], [14]. In rats undergoing spinal nerve ligation injury, Stat3 acts as a critical transducer that converts resting glia to reactive astrocytes for the maintenance of tactile allodynia [13]. Our data add to these findings in demonstrating the roles of chemokines in Stat3 regulated pain response.

Previous studies have shown that reactive astrocytes are important sources of proinflammatory mediators that regulate central sensitization during the development of pathologic pain [15]. Using cytokine antibody array, and subsequently confirmed with ELISA, a number of chemokines were identified to be up-regulated when Stat3 was activated in primary astrocytes. This finding is consistent with recent report showing that expression of Ccl20 is regulated by direct binding of Stat3 to its promoter in astrocytes [16]. In addition, we have identified putative Stat3 binding elements in the promoter regions of three other chemokines (Cxcl10, Cx3cl1 and Cxcl5). Our data have clearly established the role of Stat3 as a common transcriptional factor for a series of chemokines in reactive astrocytes. Interestingly, expression of cytokines such as IL-1β and TNFα were modestly affected, suggesting that the action of Stat3 on reactive astrocytes and pain modulation are largely dependent on the regulation of chemokine expression but not the expression of cytokines.

Chemokines from reactive astrocytes produce multiple effects in neuroinflammation. They may directly regulate the expression of proinflammatory genes in neuronal cells or indirectly by recruiting peripheral immune cells to the central nervous system [17]. Based on these mechanisms, chemokines have been shown to increase nociceptive transmission in the spinal dorsal horn [18]. Following L5 spinal nerve ligation in rats, Cx3cl1 expression was up-regulated in spinal astrocytes [19]. Subsequently, Cx3cl1 acts on microglial cells which in turn releases IL-1β and IL-6 to produce neuroinflammation and pain [20], [21]. Interestingly, mice that were devoid of specific receptors for Cx3cl1 did not exhibit mechanical allodynia following intrathecal administration a pronociceptive lysosomal cysteine protease - cathepsin S [22]. Our study has extended these findings by demonstrating Stat3 as an effective target for the modulation of Cx3cl1 level and Cx3cl1 mediated signaling. After intrathecal LPS injection, Cx3cl1 expression was up-regulated by more than 150 folds in the spinal dorsal horn. Conversely, pretreatment with Stattic reduced Cx3cl1 expression by more than 50% and attenuated LPS induced allodynia. These findings indicate that Cx3cl1 is a major contributor in Stat3 mediated nociception.

Stat3 may also modulate nociception by regulating the recruitment of peripheral immune cells. In this regard, possible candidates include Cxcl10 and Cxcl5. Recent studies reported that Cxcl10 and Cxcl5 mediated pathologic pain by recruiting neutrophil infiltration into dorsal root ganglion and dermis, respectively [23], [24]. Currently, there is no data reporting the role of Cxcl10 and Cxcl5 in the modulation of pain at the level of spinal dorsal horn. Nevertheless, infiltration of neutrophils to spinal cord has been correlated to the severity of inflammatory pain [23]. Therefore it is plausible that up-regulation of Cxcl10 and Cxcl5 may also recruit neutrophils to the spinal cord for the regulation of nociceptive transmission. In our study, we have provided *in vitro* and *in vivo* evidence that Stat3 acts as the upstream regulator of Cxcl10 and Cxcl5 that control immune cells infiltration and contribute to nociceptive transmission in the spinal cord.

Our findings are consistent with a recent report evaluating the blockade of Jak-Stat3 pathway by the overexpression of suppressor of cytokine signaling 3 (Socs3) protein. Using gliotropic lentiviral vectors in a rat model of peripheral nerve injury, Jak-Stat3 pathway was inhibited by Socs3, in a negatively feedback fashion, resulting in a decrease in neuroinflammation and mechanical allodynia [25]. However, Socs3 overexpression primarily blocks the activity of Jak2 and not on Stat3 [26]. Since Jak2 regulates other Stat proteins such as Stat6 that are also involved in the inflammatory response [27], [28], these observations could be obscured by the involvement of other STAT proteins. Therefore, Stattic was employed in our study because it is a selective Stat3 inhibitor [11]. Unlike other indirect inhibitors that block the upstream activities of Jaks, Stattic was designed as a direct blocker of Stat3 protein. This highlights the role of Stat3 in neuroinflammation.

## Conclusions

In summary, our study showed that Stat3 was activated and contributed to LPS dependent pain. In addition to the regulation of astrocytes proliferation, Stat3 also regulated the release of chemokines, especially Cx3cl1, and contributed to nociceptive transmission in the spinal cord. Stat3 therefore represents an attractive target for pain intervention.
